# Serum metabolic profiling identified a distinct metabolic signature in patients with idiopathic pulmonary fibrosis – a potential biomarker role for LysoPC

**DOI:** 10.1186/s12931-018-0714-2

**Published:** 2018-01-10

**Authors:** Barbara Rindlisbacher, Cornelia Schmid, Thomas Geiser, Cédric Bovet, Manuela Funke-Chambour

**Affiliations:** 1 University Institute of Clinical Chemistry, Inselspital, Bern University Hospital, CH-3010 Bern, Switzerland; 2 Department of Pulmonary Medicine, Inselspital, Bern University Hospital, University of Bern, Bern, Switzerland

**Keywords:** Idiopathic pulmonary fibrosis, Serum, Ultra high-performance liquid chromatography, High-resolution mass spectrometry, Metabolomics, Lysophosphatidylcholine

## Abstract

**Background:**

Idiopathic pulmonary fibrosis (IPF) is a lethal lung disease of unknown etiology. Patients present loss of lung function, dyspnea and dry cough. Diagnosis requires compatible radiologic imaging and, in undetermined cases, invasive procedures such as bronchoscopy and surgical lung biopsy. The pathophysiological mechanisms of IPF are not completely understood. Lung injury with abnormal alveolar epithelial repair is thought to be a major cause for activation of profibrotic pathways in IPF. Metabolic signatures might indicate pathological pathways involved in disease development and progression. Reliable serum biomarker would help to improve both diagnostic approach and monitoring of drug effects.

**Method:**

The global metabolic profiles measured by ultra high-performance liquid chromatography coupled to high-resolution mass spectrometry (UHPLC-HRMS) of ten stable IPF patients were compared to the ones of ten healthy participants. The results were validated in an additional study of eleven IPF patients and ten healthy controls.

**Results:**

We discovered 10 discriminative metabolic features using multivariate and univariate statistical analysis. Among them, we identified one metabolite at a retention time of 9.59 min that was two times more abundant in the serum of IPF patients compared to healthy participants. Based on its ion pattern, a lysophosphatidylcholine (LysoPC) was proposed. LysoPC is a precursor of lysophosphatidic acid (LPA) – a known mediator for lung fibrosis with its pathway currently being evaluated as new therapeutic drug target for IPF and other fibrotic diseases.

**Conclusions:**

We identified a LysoPC by UHPLC-HRMS as potential biomarker in serum of patients with IPF. Further validation studies in a larger cohort are necessary to determine its role in IPF.

**Trial Registration:**

Serum samples from IPF patients have been obtained within the clinical trial NCT02173145 at baseline and from the idiopathic interstitial pneumonia (IIP) cohort study. The study was approved by the Swiss Ethics Committee, Bern (KEK 002/14 and 246/15 or PB_2016–01524).

**Electronic supplementary material:**

The online version of this article (doi: 10.1186/s12931-018-0714-2) contains supplementary material, which is available to authorized users.

## Background

Idiopathic pulmonary fibrosis (IPF) is a severe and progressive fibrosing interstitial lung disease with sinister prognosis. The median survival is about 3–5 years after onset of symptoms, although there are various disease progression patterns [1–3]. Patients present with unspecific symptoms and signs, such as dyspnea on exertion, bibasilar inspiratory crackles and non-productive cough [4, 5]. Males over 60 years with a history of smoking have an increased risk to develop IPF. Still the exact cause remains unknown [6, 7]. Abnormal alveolar epithelial wound healing with activation of pro-fibrotic pathways is considered as a major initiating event for IPF [8]. In addition, accumulation of myofibroblasts and production of extracellular matrix leads to scarring of lung tissue with loss of function [9]. Several pro-fibrotic pathways involved in the pathogenesis of IPF have been identified and targeted for drug development [10]. Today, disease-modifying drugs are available, but none of them leads to a cure. Anti-fibrotic treatment mainly slows down disease progression [11].

IPF is diagnosed in a multidisciplinary setting with typical patterns in high resolution computed tomography (HRCT) and, if necessary, invasive methods as bronchoscopy and lung biopsy [1]. The diagnosis is made if typical criteria of usual interstitial pneumonia (UIP) pattern are fulfilled in HRCT and/ or in lung biopsy and other causes of interstitial lung diseases (ILD) have been excluded [1]. Invasive procedures are of risk for respiratory-impaired patients and not always possible. Less invasive methods are urgently needed to facilitate the diagnostic, prognostic and therapeutic monitoring strategies. Blood is easy to obtain without putting the patients at risk. Unfortunately, no serum or plasma biomarkers have been established in clinical routine so far and new biomarker candidates are urgently needed [12].

Nowadays, global metabolic profiling using ultra high-performance liquid chromatography coupled to high-resolution mass spectrometry (UHPLC-HRMS) offers the possibility to discover pathologically regulated molecules. These molecules might give information about aberrant metabolic pathways in a disease. Characteristic metabolites might offer the opportunity to understand pathological processes and to discover new potential diagnostic and/or prognostic biomarkers [13]. To our knowledge, no study investigating the global blood metabolic profile from IPF patients by UHPLC-HRMS is currently available. This approach has been recently used to identify metabotypes of asthma severity [14]. For IPF, proposed biomarkers belong to chemokines (IL-8, CCL18), proteases (MMP-1 and MMP- 7), and growth factors (IGBPs) families [15]. None of these proteins biomarkers has been established in clinical routine. In this study, we aimed to search for different metabolic profiles to identify new possible pathways and potential biomarkers for IPF.

## Methods

### Study cohort

For the pilot and validation study, serum samples of patients with stable IPF (*n* = 10, *n* = 11 respectively) and control subjects (*n* = 10, *n* = 11 respectively) were obtained from the Department for Pulmonary Medicine, University Hospital, Inselspital, Bern, Switzerland and stored by −80 °C until analysis. IPF was diagnosed according to the current international guidelines of 2011 [1]. High resolution chest CT scans of IPF patients were evaluated by a radiologist experienced in ILD diagnosis. If necessary, a histological evaluation including face-to-face multidisciplinary discussion with the treating pulmonologists, pathologists and radiologists was done. Clinical baseline characteristics, including smoking habits and lung function, were assessed and documented. Part of the serum samples from IPF patients have been obtained within the clinical trial NCT02173145 at baseline and from the idiopathic interstitial pneumonia (IIP) cohort study. The study protocol was approved by the local Ethics Committee (Swiss Ethics Committee, Bern (KEK 002/14 and 246/15 or PB_2016–01524)) and all participants signed written informed consent to participate.

### Sample preparation

Serum metabolites were extracted with organic solvent for protein precipitation. Frozen serum was gently thawed at 4 °C. 250 μL of −20 °C cold methanol:acetonitrile (1:1, v/v) containing chlorpropamide as internal standard (1 μg/mL) was added to 50 μL serum in a 1.5 mL CapLock tube. Samples were shortly vortexed, stored at −80 °C for 30 min and then centrifuged twice at 14′000 g for 20 min at 4 °C. The supernatants were transferred into total recovery liquid chromatography – mass spectrometry (LC-MS) vials. In addition to the serum samples, a blank sample was similarly extracted (water collected in a similar CapLock tube used for sample preparation). Pooled group samples were generated by combining 50 μL of each samples from the same group. A pooled quality control (QC) sample was generated by mixing 50 μL of the pooled group samples. The final serum extracts were stored at 6 °C until analysis.

### UHPLC-HRMS and TWIM-MS analysis

The serum extracts were analyzed in a randomized block design order on a high resolution mass spectrometer (Synapt G2-S HDMS, Waters, Milford, MA, USA) coupled to a 2D UPLC Acquity I-Class system (Waters, Milford, MA, USA). Two μL of the extracted metabolites were separated on a ACQUITY UPLC HSS T3 column (1.0 mm × 100 mm, 1.8 μm, Waters) at a flow rate of 0.17 mL/min. The mobile phase was composed of 0.1% (v/v) formic acid in (A) 1% (v/v) methanol in H_2_O and (B) methanol. The following LC gradient was applied: 0 min: 100% mobile phase A, 1 min: 100% A, 11 min: 1% A, 13.0 min: 1% A, 13.1 min: 100% A, 15 min: 100% A. The column temperature was set to 50 °C and the autosampler to 6 °C. Standard mass spectrometric parameters were 0.5 kV and 20 V for capillary voltage and cone voltage, respectively. Desolvation and source temperature were kept at 450 °C and 120 °C, respectively. Cone and desolvation flows were set to 150 L/h and 800 L/h, respectively. Leucine-Enkephalin ([M + H]^+^*m/z* = 556.2766, [M-H]^−^*m/z* = 554.2620) was acquired every 20 s for lock mass correction. Mass spectra were acquired at a scan time of 0.2 s in the MS^E^ resolution mode between *m/z* 50 and 1200. In addition, data-dependent acquisition (DDA) MS/MS experiments were performed on the pooled group and QC samples. Before sample analysis, the system was equilibrated by injecting 10 times the QC sample and the QC sample was then analyzed after every fifth sample injection. A system suitability test (SST) containing standards was measured at the beginning and the end of the analytical run to ensure retention time stability, intensity stability and mass error ≤ 8 ppm. The instrument was controlled via MassLynx (version 4.1, Waters). The pilot study set was analyzed with one replicate per IPF patient and healthy control sample, whereas for the validation set, duplicate analysis was applied.

To improve structural elucidation of the regulated metabolites, traveling wave ion mobility mass spectrometry (TWIM-MS) of extracted serum was additionally performed. Extracted serum was separated under the same chromatographic conditions described above. Mass spectra were acquired in the positive and negative ionization mode at a scan time of 0.5 s in the HDMS^E^ resolution mode between *m/z* 50 and 1200. The TWIM wave height was set at 40 V and the wave velocity was ramped from 1000 to 300 m/s. Precursors were fragmented after the TWIM separation in the transfer T-cell set and transferred to the TOF at a velocity of 220 m/s. The ion mobility was calibrated with poly-DL-alanine, which allowed the determination of the collision cross section (CCS) in nitrogen [16]. A lipid standard mixture of phosphatidylcholines (PC(18:0/0:0), PC(18:2/0:0), PC(12:0/12:0), PC(14:0/14:0), PC(18:1/18:1), PC(16:0/0:0) obtained from Echelon Biosciences) was prepared in acetonitrile:isopropanol:water (2:1:1, v/v) and used as quality control for the CCS measurements. Two μL of this lipid standard mixture at 0.1 and 1.0 μg/mL were analyzed by UHPLC-TWIM-MS in the positive and negative ESI mode, respectively. Data processing was performed using UNIFI (version 1.8.2.169, Waters, Millford, USA).

### Data processing and statistical analysis

After lock-mass correction and chromatographic alignment with Progenesis QI (version 2.2, Nonlinear Dynamics, Newcastle, UK), ion patterns were deconvoluted between 0.45–11.5 min. As possible ions, [M + H]^+^, [M + Na]^+^, [M + K]^+^, [M + H-H_2_O]^+^, [M + H-2H_2_O]^+^, [M + 2H]^2+^, [M + 3H]^3+^, [2M + H]^+^, [2 M + Na]^+^ and [2M + K]^+^ were defined in the positive ion mode, [M-H]^−^, [M-H-H_2_O]^−^, [M + HCOOH-H]^−^, [M-2H]^2−^, [M-3H]^3−^ and [2M-H]^−^ in the negative ion mode. The abundances of the features were normalized to all compounds. Features eluting between 0.5–10.5 min with a chromatographic peak width ≥ 0.05 min, having a coefficient of variation of the peak area ≤ 30% in the QC samples, singly charged ions and ions having a maximum abundance across all samples ≥200 (positive mode, arbitrary threshold) or ≥150 (negative mode, arbitrary threshold), were selected for further evaluation. In addition, the metabolic features having an abundance in blank higher than in QC and which were at least twice more abundant in blank were excluded. Known drug/drug metabolites taken from patient were also excluded from the dataset.

The normalized abundances of each filtered metabolic features were subjected to multivariate and univariate statistical analysis. For the validation set, the features abundances of the duplicate analysis were averaged. For multivariate statistical analysis, data were subjected to Pareto scaling prior to orthogonal projections of latent structures discriminant analysis (OPLS-DA) with SIMCA (version 14, Umetrics, Umeå, Sweden). Results were further integrated if the n-fold (n = total number of sample, leave-one-out cross validation approach) cross-validated correlation Q^2^(cum) was ≥0.5, if the cross validatory ANOVA *p*-value was ≤0.05 and if the permutation test was passed (20 permutations, R^2^ > R^2^_permutation_ and Q^2^_permutation_ < 0). Discriminative features were selected based on a VIP_PRED_ score ≥ 1.5 and absolute p (corr) ≥ 0.5. The confidence interval of the selected features was also not including 0. With this approach, only metabolic features with strong model contribution and high reliability were selected. Univariate statistical analysis was performed to consistently evaluate the filtered metabolic features and to screen for additional metabolites. For the pilot study, univariate analysis of variance (ANOVA) was based on arcsinh transformed data from Progenesis QI. For the validation study, ANOVA was performed with SIMCA and was based on log2 transformed and Pareto scaled normalized abundances. The false discovery rate (FDR) due to multiple testing was estimated according to Benjamini Hochberg [17]. FDR corrected *p*-values (i.e. q-value) threshold was set at q ≤ 0.05.

Structure and formula of the isolated discriminative metabolic features were searched against the Human Metabolome Database (HMDB, version 3.6) and LIPID MAPS Structure Database (LMSD, version from December 6, 2016) with a mass accuracy of 8 ppm. Metabolic features with only one adduct ion were assumed to be [M + H]^+^ and [M-H]^−^ ions in the positive and negative ionization mode, respectively.

## Results

### Study cohort

In the pilot study, 10 IPF patients and 10 age- and sex-matched healthy controls were included. The mean age of IPF patients and of healthy controls was 67.8 respectively 68.7 years. Ninety percent of all participants were male in both groups. Lung functional measurements showed a reduced median forced vital capacity (FVC) of 2.4 L (mean 65% predicted) in IPF patients and a median FVC of 4.1 L (mean 115% predicted) in controls. Average 3-year mortality prediction in IPF patients was in 42.1% according to the currently used GAP staging method (stage II) [2]. Eighty percent of IPF patients and controls had a history of cigarette smoking.

For the validation study, blood sampling from 7 IPF patients was performed 2 weeks after the initial pilot study. Four recently diagnosed IPF patients and all healthy controls were newly collected for the validation study. One sample of the control group was excluded due to analytical problems during sample preparation. Eleven IPF patients and 10 healthy controls remained for further analysis. The baseline characteristics of patients and controls of both pilot and validation study are summarized in Table 1.

**Table 1:** Baseline characteristics of healthy controls and IPF patients

	Pilot study		Validation study	
	Healthy	IPF	Healthy	IPF
Subjects (male %)	10 (90)	10 (90)	10 (60)	11 (82)
Age (years)	68.7 ± 7.9	67.8 ± 8.6	32.4 ± 15.3	66.1 ± 8.6
BMI kg/m^2^	28.9 ± 5.1	26.9 ± 3.6	22.6 ± 2.7	26.3 ± 4.3
BMI ≥ 25 kg/m^2^	7	5	2	6
Diabetes mellitus type 2 (n)	1	2	0	3
Hypertension	5	5	0	3
Lung function				
FVC, L	4.1 ± 0.4	2.4 ± 0.5	4.5 ± 1.0	2.4 ± 0.6
FVC (% predicted)	114.6 ± 8.5	65.1 ± 11.3	94.0 ± 10.3	63.1 ± 14.3
DLCO (% predicted)	129.6 ± 31.1	43 ± 15.0	87.8 ± 11.3	44.6 ± 14.6
GAP Stage (I-III)	–	I - II	–	I- II
Smoking status				
Current Smokers n (median pack years ± SD)	0	0	5 (8.2 ± 14.5)	0
Former Smokers n (median pack years ± SD)	8 (5.5 ± 5.7)	8 (25.4 ± 15.2)	0	8 (13.9 ± 13.3)
Antifibrotic therapy				
Pirfenidone n (%)	–	6 (60)	–	6 (54.5)
Nintedanib n (%)	–	3 (30)	–	4 (36.4)
No antifibrotics n (%)	–	1 (10)	–	1 (9.1)

*FVC* Forced vital capacity, *DLCO* Diffusing capacity of the lung for carbon monoxide, *BMI* Body Mass Index

### Metabolic serum profiling by UHPLC-HRMS

**Fig. 1:**
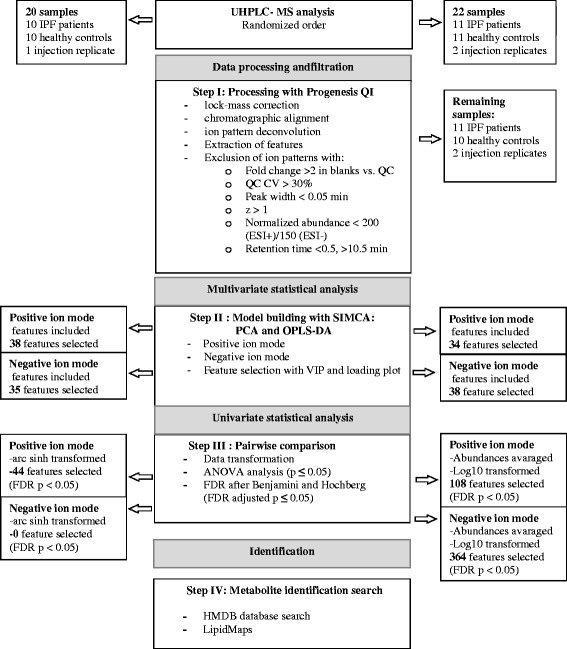
Flowchart for sample measurement, data processing and statistical analysis. [60]

**Fig. 2:**
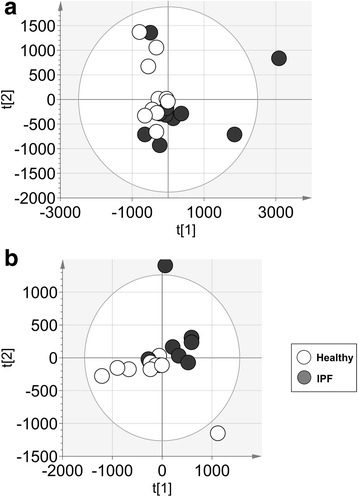
PCA score plots of the healthy participants and IPF patients of the pilot study measured in the (**a**) positive and (**b**) negative ESI mode

The differentially regulated serum metabolites in healthy participants and IPF patients were searched for by non-targeted metabolic profiling using UHPLC-HRMS. The flowchart for sample preparation and analysis is illustrated in Fig. 1. After data processing and filtration, 2426 and 1740 metabolic features were detected in the pilot study by UHPLC-HRMS in the positive and negative electrospray ionization (ESI) mode, respectively. Principal component analysis (PCA) of the non-targeted metabolic profiles measured by UHPLC-HRMS suggested differences between healthy controls and IPF patients (Fig. 2). In the negative ESI mode, one IPF sample was defined as outlier and excluded from further statistical analysis, otherwise multivariate and univariate statistical analysis of the data did not isolate differentially regulated metabolic features (data not shown). Acceptable OPLS-DA model characteristics were obtained (Table 2) and based on the selection criteria, 67 and 103 discriminative features were selected by univariate and/or multivariate analysis in the positive and negative ESI mode, respectively (data not shown).

**Table 2:** Characteristics of the OPLS-DA models for the pilot and validation study sets analyzed in positive (ESI+) and negative (ESI-) ESI mode

Study	Ionisation	Number of samples	R^2^(cum)	Q^2^(cum)	CV ANOVA p-value	Permutation test
Pilot	ESI+	20	0.894	0.599	0.0058	R^2^ = 0.734 Q^2^ = −0.209
Validation	ESI+	21	0.903	0.742	0.00014	R^2^ = 0.694 Q^2^ = −0.318
Pilot	ESI-	19	0.872	0.503	0.0339	R^2^ = 0.767 Q^2^ = −0.299
Validation	ESI-	21	0.878	0.721	0.0003	R^2^ = 0.66 Q^2^ = −0.447

**Fig. 3:**
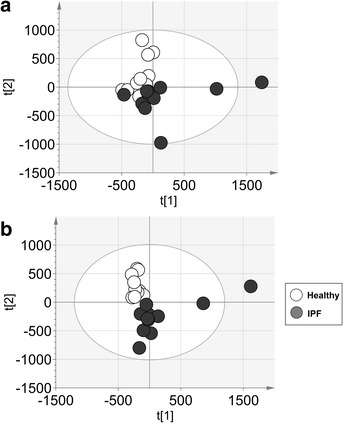
PCA score plots of the healthy participants and IPF patients of the validation study measured in the (**a**) positive and (**b**) negative ESI mode

To validate our results, a new set of samples was profiled by UHPLC-HRMS. After data processing and filtration, 1229 and 1059 metabolic features were detected by UHPLC-HRMS in the positive and negative ESI mode, respectively. Based on the PCA score plots (data not shown), one healthy participant was considered as outlier and was further excluded from statistical analysis. The PCA score plots obtained after data filtration and exclusion of this participant are shown in Fig. 3. Similar to the pilot study, satisfactory multivariate model parameters were obtained in both ionization modes (Table 2). Based on our selection criteria, 103 and 364 discriminative features were selected by univariate and/or multivariate analysis in the positive and negative ESI mode, respectively (data not shown).

To identify common metabolic features regulated between healthy participants and IPF patients in the pilot and validation study, the retention time and *m/z* ratio of the discriminative metabolic features isolated in each study by multivariate and/or univariate analysis were compared. Among them, 3 and 27 metabolic features were commonly found in the positive and negative ionization mode, respectively. Based on their abundance profile (Additional file 1: Figure S1), we hypothesized that the metabolic features mainly detected in the IPF samples were related to drugs and/or drug metabolites. After exclusion of these features, 10 metabolic features remained differentially regulated in the pilot and were confirmed in the validation study. Their corresponding fold changes in abundance and potential annotations found against the metabolite databases are summarized in Table 3.

**Table 3:** LC-MS characteristics (retention time, *m/z* ratio) and fold changes of the metabolic features isolated by multivariate and/or univariate analysis (FDR ≤ 0.05) in the pilot and validation study

Study	Compound ID	Potential ID [adduct]	Ionisation	*m/z*	RT (min)	Fold change IPF/Healthy	FDR (Univariate)	Multivariate
Pilot Validation	5.94_332.2423m/z 5.94_332.2419m/z	3-hydroxydecanoyl carnitine [M+H]^+^	ESI+	332.2423 332.2419	5.94 5.94	4.0 25.9	0.047 0.003	no no
Pilot Validation	9.59_511.3269n 9.59_511.3259n	LysoPC [M+H]^+^, [M+Na]^+^, [M+K]^+^	ESI+	512.3342 512.3332	9.59 9.59	1.9 1.9	0.037 0.008	yes yes
Pilot Validation	9.59_496.3026m/z 9.58_496.3029m/z	LysoPC [M-CH_3_]^-^	ESI-	496.3026 496.3029	9.59 9.59	1.6 2.0	0.11 4.87 10^−4^	yes yes
Pilot Validation	9.59_556.3248m/z 9.59_556.3245m/z	LysoPC [M+CHOO]^-^	ESI-	556.3248 556.3245	9.59 9.59	1.9 2.1	0.048 5.19 10^−4^	yes yes
Pilot Validation	2.43_267.0728m/z 2.38_267.0737m/z	Inosine [M-H]^-^	ESI-	267.0728 267.0737	2.43 2.38	1.8 0.7	0.22 0.044	yes yes
Pilot Validation	8.77_370.1807n 8.38_369.1738m/z	Androsterone sulfate [M-H]^−^	ESI-	369.1734 369.1738	8.77 8.38	0.4 0.3	0.22 0.03	yes yes
Pilot Validation	0.88_195.8107m/z 0.65_195.8115m/z		ESI-	195.8107 195.8115	0.88 0.65	0.8 0.9	0.25 0.02	yes yes
Pilot Validation	0.88_197.8076m/z 0.65_197.8090m/z		ESI-	197.8076 197.8090	0.88 0.65	0.8 0.9	0.23 0.02	yes yes
Pilot Validation	0.88_199.8045m/z 0.65_199.8057m/z		ESI-	199.8045 199.8057	0.88 0.65	0.8 0.9	0.24 0.03	yes yes
Pilot Validation	8.57_371.1879m/z 8.57_371.1879m/z		ESI-	371.1879 371.1889	8.57 8.60	0.3 0.2	0.24 0.04	yes no

The identity of inosine was confirmed by comparing the retention times and fragmentation patterns obtained in serum to an authentic inosine standard. This purine metabolite was upregulated in the pilot study and downregulated in the validation study. Only three metabolic features eluting at 5.94 min (*m/z* = 332.242) and 9.59 min (*m/z* = 512.334 in the positive ESI mode and *m/z* = 556.325 in the negative ESI mode) were significantly upregulated in IPF patients in both studies.

The metabolic features eluting at 5.94 min was potentially assigned against the HMDB database to 3-hydroxydecanoyl carnitine (chemical formula C_17_H_33_NO_5,_ mass error − 2.7 ppm, isotope similarity 96%). The detection of the characteristic acylcarnitine fragment [C_4_H_5_O_2_]^+^ at *m/z* 85.0284 was confirmed by UHPLC-TWIM-MS measurement (mass error − 4.8 ppm, Additional file 2: Figure S2). No significant differences were found in the global acylcarnitine profile (data not shown, profile extracted as in Bally et al. [18]).

**Scheme 1:**
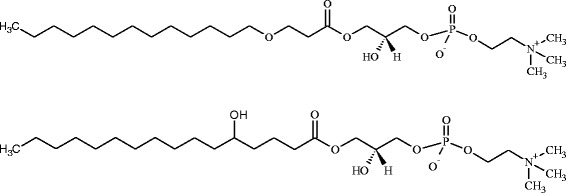
Potential structures of the LysoPC which was elevated in serum of IPF patients compared to controls. For simplification, only the sn-1 isomers are represented

**Fig. 4:**
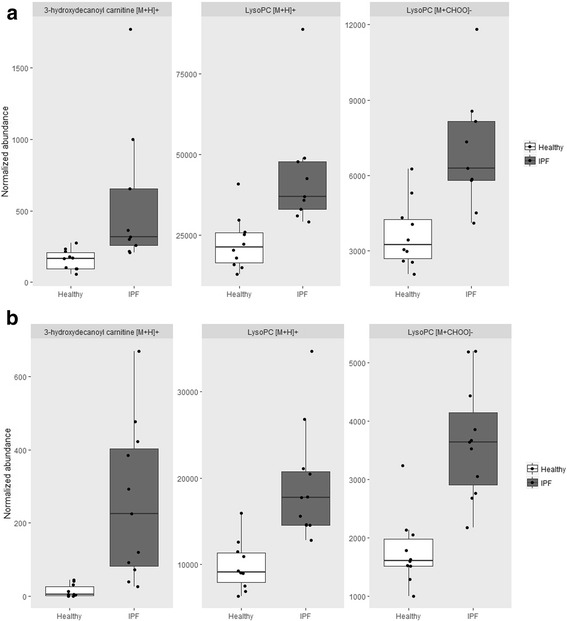
Normalized abundances of the common significantly regulated metabolic features detected in the (**a**) pilot study and (**b**) validation study. The metabolic feature eluting at 5.94 min was assigned to 3-hydroxydecanoyl carnitine ([M + H]^+^, *m/z* = 332.242), the ones eluting at 9.59 min to the LysoPC (neutral mass 511.327 amu, and [M + CHOO]^−^ at *m/z* = 556.325)

At 9.59 min, three discriminative metabolic features were two times more abundant in the serum of IPF patients compared to healthy participants (Table 3, *m/z* = 512.334 in the positive ESI mode, *m/z* = 556.325 and 496.303 in the negative ESI mode, with the latter being significant only in one study). Presumably, these co-eluting ions belonged to the same metabolic features. In the negative ESI mode, the detected ion pattern agreed with a phosphatidylcholine (PC) or sphingomyelin [M-CH_3_]^−^ and [M + CHOO]^−^ ions. These ion patterns were confirmed by the analysis of PC lipid standards (data not shown) and agreed with the literature [19, 20]. The MS/MS spectra acquired in the positive and negative ESI mode (DDA experiments, Additional file 3: Figure S3**)** further suggested the detection of a LysoPC with the chemical formula C_24_H_50_NO_8_P (mass error − 1.0 ppm, isotope similarity 98%). No LysoPC structure matching the MS data was found against the LIPID MAPS database and the literature. Therefore, we assumed a LysoPC structure with an ether or hydroxylated acyl chain in the sn-1 or sn-2 position (Scheme 1). We further characterized this unknown LysoPC with TWIM-MS and measured a CCS of 227.5 Å^2^ for the protonated ion (Table 4). Excellent CCS agreements with the literature (Table 4, CCS error < 1.1%) were obtained for the PC standard mixture confirming the excellent inter-laboratory reproducibility of CCS values [16]. A single TWIM peak was detected for the unknown LysoPC, which suggested the detection of a single LysoPC having a compacter conformation than PC(16:0/0:0). To our knowledge, the influence of ether or hydroxylated acyl chain on the LysoPC CCS has not been reported. It is therefore difficult to hypothesize the most probable structure for this unknown LysoPC based on the TWIM-MS measurement. The abundance profiles of all significantly upregulated metabolic features detected in the pilot and validation study (3-hydroxydecanoyl carnitine, and 2 metabolic features of LysoPC) are shown in Fig. 4.

**Table 4:** CCS values obtained from the ion mobility measurements of the lipid standards and the unknown LysoPC. Results shown are averages from three sample analysis

Component [M + H]+	Formula	Average RT (min)	Average CCS (Å^2^)	RSD CCS (%)	Literature CCS (Å^2^)	CCS error (%)
Unknown LysoPC	C24H50NO8P	9.55	227.5	0.0		
PC(16:0/0:0)	C24H50NO7P	11.01	237.9	0.2	236 [16]	0.8
PC(18:2/0:0)	C26H50NO7P	10.83	234.7	0.1		
PC(18:0/0:0)	C26H54NO7P	11.46	245.2	0.1	246 [16]	1.1
PC(12:0/12:0)	C32H64NO8P	11.96	269.4	0.1	272.4 [19]	−1.1
PC(14:0/14:0)	C36H72NO8P	12.52	281.9	0.2	282.9 [19]	−0.4
PC(18:1/18:1)	C44H84NO8P	13.38	300.6	0.2		

*CCS* Collision cross section

## Discussion

After excluding potential drugs and drug metabolites, ten metabolic features were isolated in serum from IPF patients by non-targeted UHPLC-HRMS based metabolomics. Among them, 3 metabolic features were significantly upregulated in IPF patients in both, pilot and validation study. Based on the MS data and the databases, we hypothesized the detection of 3-hydroxydecanoyl carnitine and a LysoPC, the second as a potential precursor for lysophosphatidic acid (LPA) [21].

Identification of differences between IPF and healthy individuals by biomarker is important for diagnosis and prognosis and supports the clinician for evaluation of individual treatment responses. In addition, heterogeneity of disease course suggests different pathophysiological phenotypes in IPF. A recent study identified molecules of the autotaxin-lysoPA pathway by metabolomics analysis equally for patients with COPD [22]. In this study, lung functional parameters in males were associated with LPA subspecies levels in COPD [22]. Due to the predominant male patients and small number of participants we are unable to further investigate a gender association in our study.

In the positive ESI mode, we detected a metabolic feature (*m/z* 332.242 at a retention time of 5.94 min) with possible identification of 3-hydroxydecanoyl carnitine, a medium-chain acylcarnitine, which was upregulated in serum of IPF patients (pilot FC 4.0, validation FC 25.9). We did not detect sufficient hydroxycarnitines species to further evaluate their profile because of the insufficient sensitivity of our method. In addition, no significant differences were found in the global acylcarnitine profile. In contrast, medium-chain acylcarnitines were decreased in lung tissue from IPF patients in a recent metabolomics study [23]. According to the authors this may be due to an impaired transport of fatty acids into the mitochondria [23].

A metabolic profiling study using gas-chromatography coupled to mass spectrometry of IPF lung tissue showed that the purine metabolites inosine and hypoxanthine were increased compared to normal lung tissue [24]. Inosine is the precursor for adenosine. In experimental models, adenosine levels have been associated with disease progression for lung fibrosis and could be abrogated by adenosine inhibitor dipyridamole [25]. Extracellular adenosine levels are associated with the progression and exacerbation of pulmonary fibrosis [25]. In addition, adenosine receptors were increased in lungs form IPF patients [26]. Purinergic signaling and scarring of fibrotic tissue have been largely associated [27]. Interestingly, in our study, we found a discordant regulation of inosine between the pilot and the validation set. While in the age-matched pilot study serum levels of inosine were increased in IPF patients, the validation study showed an opposing result. A possible explanation is the lack of age-matched validation cohort. Age-dependent serum adenosine deaminase activity may have had an impact on this result [28].

In our study, we consistently identified a LysoPC in serum from IPF patients. LysoPC is a precursor of LPA, which is a bioactive glycerophospholipid. There are multiple LPA types differing in length and degree of saturation of the fatty acid [29]. LPA is involved in various processes as fibrosis, systemic sclerosis, cancer, inflammation, atherosclerosis, obesity, asthma, multiple sclerosis, neuropathic pain or embryonal implantation [30–39]. LPA induces fibrosis in the lung, kidney and liver by epithelial cell death, vascular leakage and fibroblast migration and proliferation [30, 40–42].

The wide range of biological effects can be explained by multiple LPA receptors. LPA mediates its responses via at least six LPA receptors [21]. Pro-fibrotic effects are induced by activation of the specific LPA receptor LPA_1_ [21]. Both genetic suppression of LPA-receptor 1 and antagonism of the enzyme autotaxin prevent pulmonary fibrosis [42–44]. LPA_1_-deficient mice did not develop pulmonary fibrosis after bleomycin injury [42]. Additionally, LPA via LPA_2_ induces αvβ6 integrin-mediated transforming growth factor-β (TGF-β) activation in bronchial epithelial cells [45]. It was shown in another study, that LPA_2_ knockout mice did not develop pulmonary fibrosis after bleomycin application. Moreover, the expression of TGF-β in human lung fibroblasts was inhibited [46].

The source of LPA is unclear. There are at least two different sources of LPA production. One is the hydrolysis of phosphatidic acids, which are located in cell membranes, by phospholipase A1 and A2. But the majority of LPA is produced by the enzymatic cleavage of LysoPC and lysophosphatidylserine by lysophospholipase D activity of the enzyme autotaxin (ATX) [47–49]. In IPF, epithelial cell injury, specifically of type 2 alveolar epithelial cells, is thought to initiate the fibrotic process [9]. After lung injury, dipalmitoylphosphatidylcholine (DPPC), as major surfactant lipid component, is degraded to LysoPC by phospholipase A2 activity by type 2 alveolar epithelial cells [50]. As explained above, LysoPC is a precursor of LPA and converted by the enzyme autotaxin (ATX) [21]. LPA via LPA_1_ promotes epithelial cell apoptosis after lung injury [51]. But also platelet activation can lead to LPA via autotaxin [52]. In IPF a prothrombotic state is recognized and platelet activation is increased compared to controls [53]. Increased platelet activation might contribute to elevated LPA levels in IPF. Previous studies showed increased level of LPA in the bronchoalveolar lavage fluid and exhaled breath condensate of IPF patients [42, 54].

In addition, COPD metabolomics studies also revealed alteration of LPA pathway [22]. LPA pathway has been associated with several different lung disease including hyperoxic lung injury and asthma [36, 55, 56]. As therapeutic approach to interrupt the LysoPC - LPA axis, an oral inhibitor of autotaxin (GLPG1690) is currently evaluated in a Phase 2 study for IPF patients [57, 58]. If the production of LPA is reduced by inhibition of autotaxin, both LPA_1_ and LPA_2_ pathways would be targeted [58]. In our study, LPA was not detectable in our samples due to the analytical method. However, we were able to show upregulated LysoPC in serum of IPF patients, indicating the crucial role of LysoPC-LPA pathway in IPF. Now, our findings need to be confirmed by different methods in a larger cohort of IPF patients including correlation to clinical parameters and disease severity.

## Conclusions

In our study, we detected by global UHPLC-HRMS based metabolic profiling an increased serum level of a potential LysoPC and 3-hydroxydecanoyl carnitine in IPF patients compared to healthy controls. If confirmed in a larger cohort, the LysoPC might represent a potential marker for diagnostic and monitoring of IPF. The clinical meaning of this potential metabolite and its significance need to be evaluated in future studies. Global metabolic profiling by UHPLC-HRMS is a promising approach to detect possibly relevant pathophysiological pathways and potential serum biomarkers in IPF.

## Additional files

Additional file 1: Figure S1. Heat maps representing the log10-transformed abundance profile (Pareto scaled) of the regulated metabolic features isolated by multivariate and/or univariate statistical analysis in the (top) pilot study and (bottom) validation study. Identity (summarized by the retention time followed by the corresponding *m/z* ratio or neutral mass n) of the metabolic features are shown on the right side. Cells colored in red represent up-regulated, colored in blue down-regulated abundances. The analysis was done with the MetaboAnalyst online platform [59]. (PNG 581 kb)

Additional file 2: Figure S2. MS (top, HDMS^E^ experiment) and MS/MS spectra (bottom, HDMS^E^ experiment) of the metabolic features eluting at 5.9 min acquired in the positive ESI mode and potentially assigned to the [M + H]^+^ 3-hydroxydecanoyl carnitine ion. The detection of the characteristic acylcarnitine fragment [C_4_H_5_O_2_]^+^ at *m/z* 85.0284 was confirmed by MS/MS spectra (bottom, HDMS^E^ experiment). (PDF 99 kb)

Additional file 3: Figure S3. MS/MS spectra (DDA experiments) of the metabolic features eluting at 9.6 min acquired in the (a) positive and (b) negative ESI mode and potentially assigned to the [M + H]^+^ and [M + CHOO]^−^ LysoPC ions, respectively. The main fragments are annotated with the corresponding structure, measured *m/z* ratio and mass accuracy. (PDF 150 kb)

## Abbreviations

CCS: Collision cross section
DLCO: Diffusing capacity of the lung for carbon monoxide
ESI: Electrospray ionization
FDR: False discovery rate
FVC: Forced vital capacity
HDMS^E^: High definition mass spectrometry
HMDB: Human metabolome database
HRCT: High resolution computed tomography
HRMS: High-resolution mass spectromety
ILD: Interstitial lung disease
IPF: Idiopathic pulmonary fibrosis
LPA: Lysophosphatidic acid
LysoPC: Lysophosphatidylcholine
MS: Mass spectrometry
OPLS-DA: Orthogonal partial least squares discriminant analysis
PCA: Principal component analysis
TGF- β: Transforming growth factor-β
TWIM: Traveling wave ion mobility
UHPLC: Ultra high-performance liquid chromatography
UIP: Usual interstitial pneumonia
VIP: Variable influence on projection
