# Supplementary figures and images for: Serum metabolic profiling identified a distinct metabolic signature in patients with idiopathic pulmonary fibrosis – a potential biomarker role for LysoPC
Source: Respir Res. 2018 Jan 10;19:7. doi: 10.1186/s12931-018-0714-2 (PMC5764001; doi:10.1186/s12931-018-0714-2)

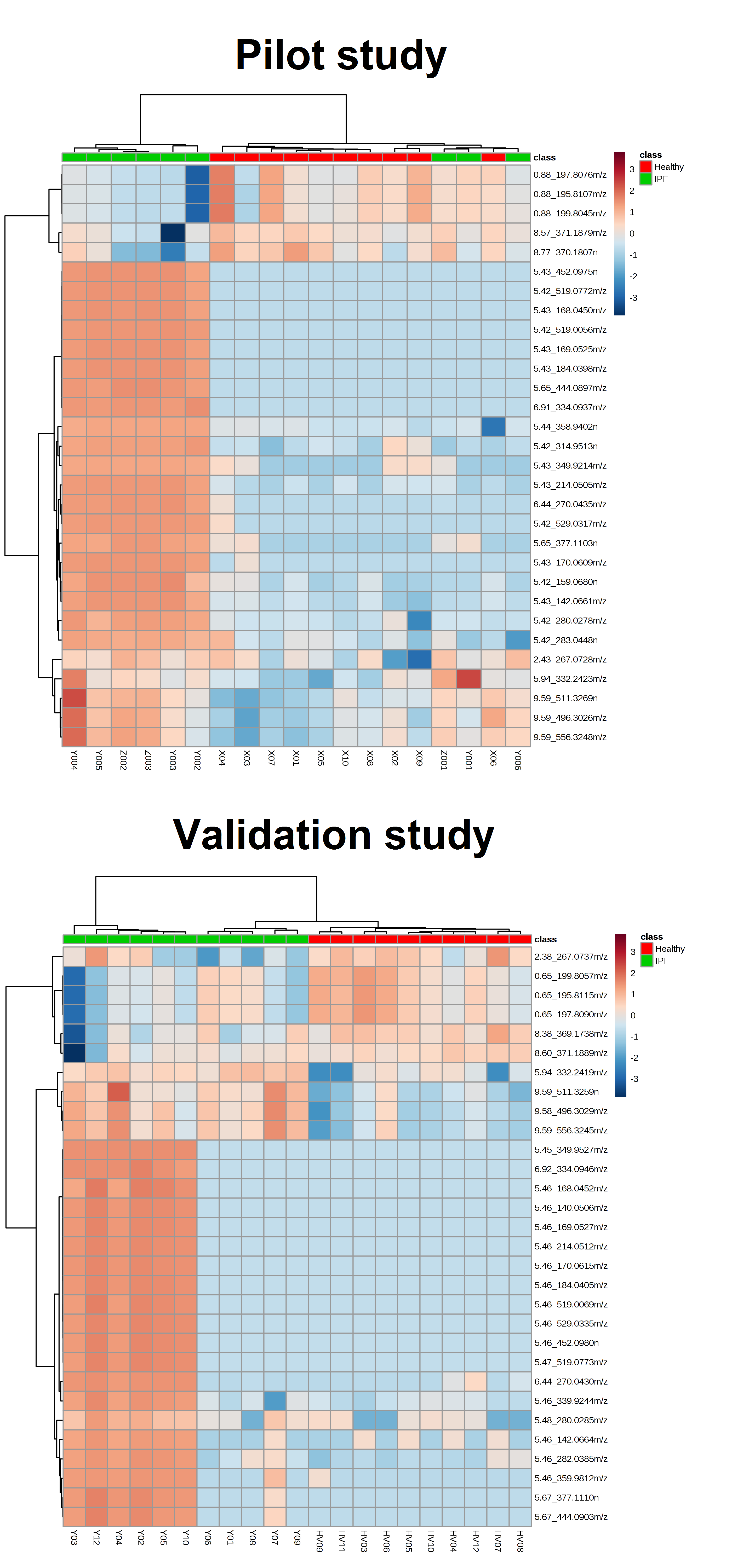

Supplement: Supplementary file 1 — Heat maps representing the log10-transformed abundance profile (Pareto scaled) of the regulated metabolic features isolated by multivariate and/or univariate statistical analysis in the (top) pilot study and (bottom) validation study. Identity (summarized by the retention time followed by the corresponding m/z ratio or neutral mass n) of the metabolic features are shown on the right side. Cells colored in red represent up-regulated, colored in blue down-regulated abundances. The analysis was done with the MetaboAnalyst online platform [59]. (PNG 581 kb) [file 12931_2018_714_MOESM1_ESM.png]

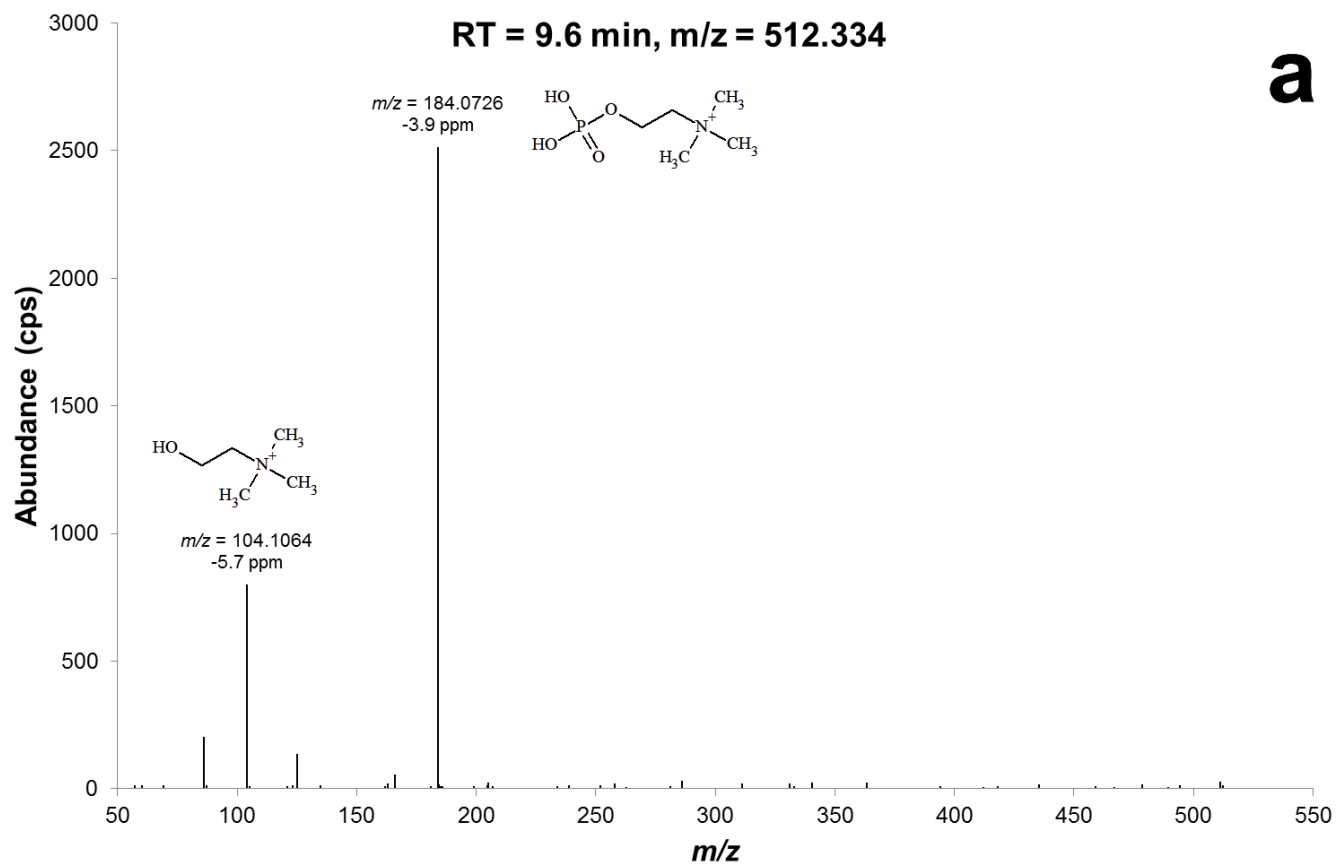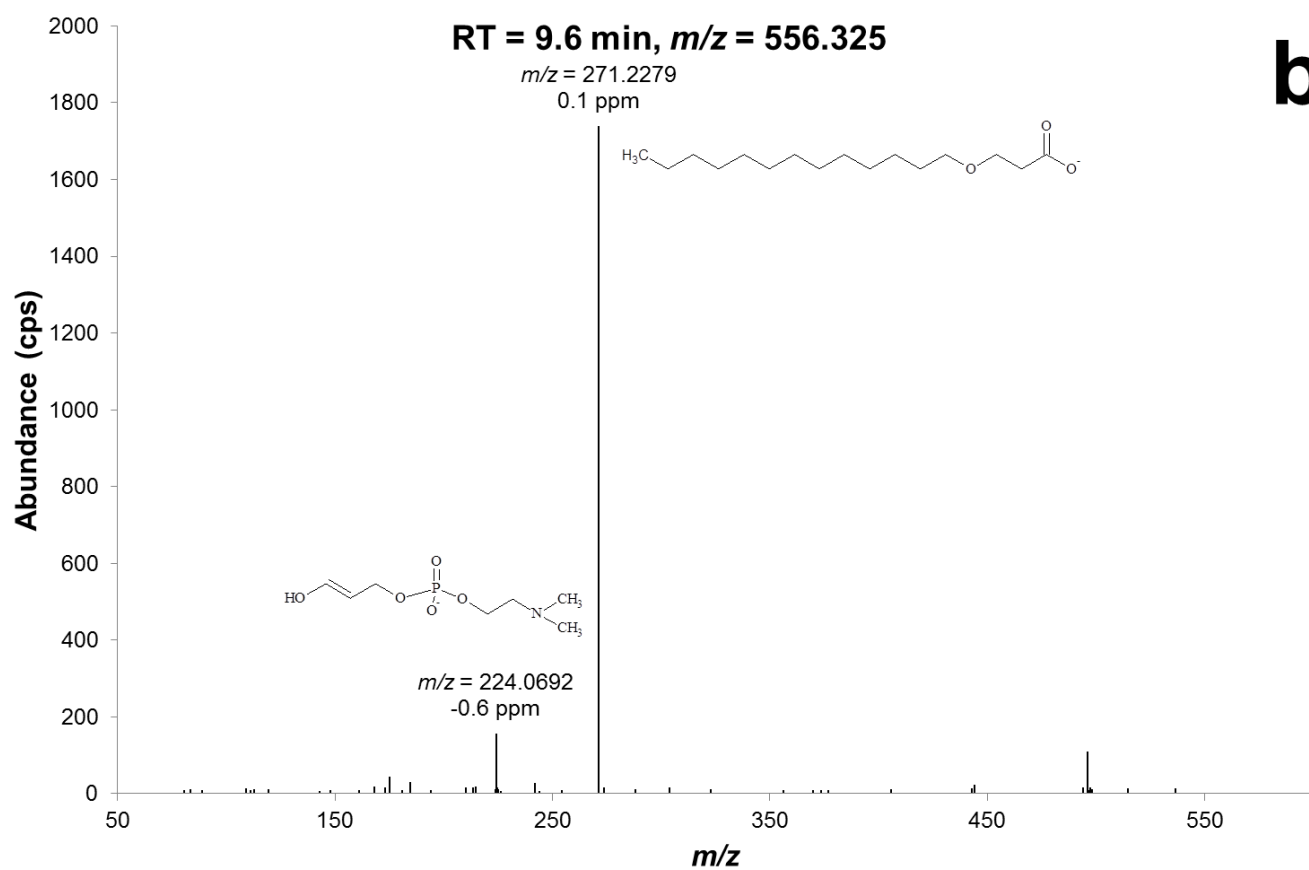

Supplement: Supplementary file 3 — MS/MS spectra (DDA experiments) of the metabolic features eluting at 9.6 min acquired in the (a) positive and (b) negative ESI mode and potentially assigned to the [M + H]+ and [M + CHOO]− LysoPC ions, respectively. The main fragments are annotated with the corresponding structure, measured m/z ratio and mass accuracy. (PDF 150 kb) [file 12931_2018_714_MOESM3_ESM.pdf]
