# Supplementary material for: Serum metabolic profiling identified a distinct metabolic signature in patients with idiopathic pulmonary fibrosis – a potential biomarker role for LysoPC
Source: Respir Res. 2018 Jan 10;19:7. doi: 10.1186/s12931-018-0714-2 (PMC5764001; doi:10.1186/s12931-018-0714-2)

Item name: 20171115_p009_CCSSerum_HDMSe_pos_020 Channel name: Low energy : Time 5.9996 +/- 0.0552 minutes...
Item description: Exctracted Y001 Serum

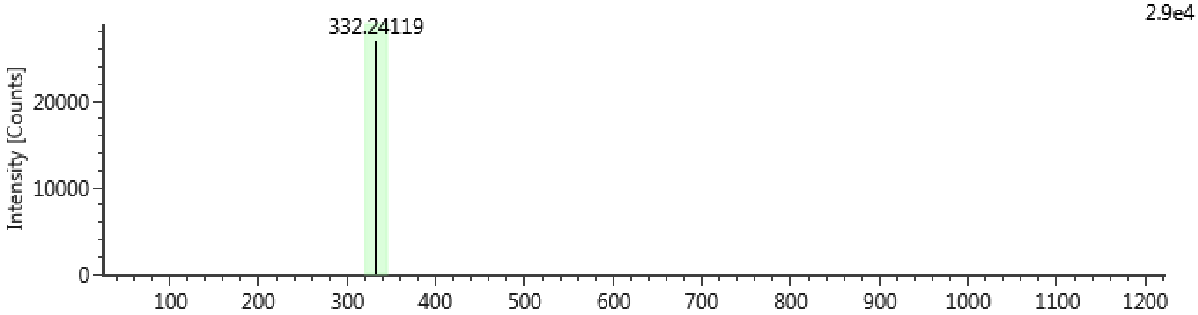

Item name: 20171115_p009_CCSSerum_HDMSe_pos_020 Channel name: High energy : Time 5.9996 +/- 0.0552 minutes...
Item description: Exctracted Y001 Serum

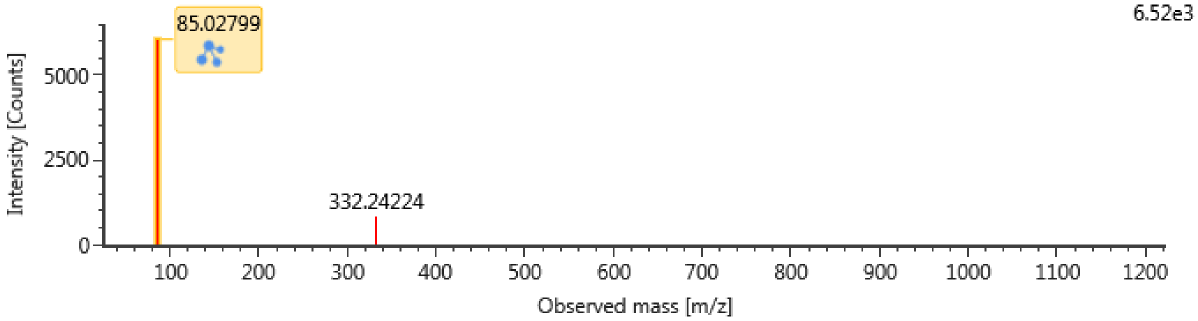

Supplement: Supplementary file 2 — MS (top, HDMSE experiment) and MS/MS spectra (bottom, HDMSE experiment) of the metabolic features eluting at 5.9 min acquired in the positive ESI mode and potentially assigned to the [M + H]+ 3-hydroxydecanoyl carnitine ion. The detection of the characteristic acylcarnitine fragment [C4H5O2]+ at m/z 85.0284 was confirmed by MS/MS spectra (bottom, HDMSE experiment). (PDF 99 kb) [file 12931_2018_714_MOESM2_ESM.pdf]
